# Lactic Acid Salts of Nicotine Potentiate the Transfer of Toxic Metals into Electronic Cigarette Aerosols

**DOI:** 10.3390/toxics12010065

**Published:** 2024-01-13

**Authors:** R. Steven Pappas, Naudia Gray, Mary Halstead, Clifford H. Watson

**Affiliations:** Centers for Disease Control and Prevention, Tobacco and Volatiles Branch, 4770 Buford Hwy, MS S110-4, Atlanta, GA 30341, USA; nrgray@cdc.gov (N.G.); cow1@cdc.gov (C.H.W.)

**Keywords:** metals, vaping, aerosol, nicotine, lactic acid, benzoic acid, ENDS, e-cigarettes

## Abstract

The designs and liquid formulations of Electronic Nicotine Delivery System (ENDS) devices continue to rapidly evolve. Thus, it is important to monitor and characterize ENDS aerosols for changes in toxic constituents. Many ENDS liquid formulations now include the addition of organic acids in a 1 to 1 molar ratio with nicotine. Metal concentrations in aerosols produced by ENDS devices with different nicotine salt formulations were analyzed. Aerosols from devices containing lactic acid had higher nickel, zinc, copper, and chromium concentrations than aerosols produced by devices containing benzoic acid or levulinic acid. Our scanning electron microscope with energy dispersive X-ray analytical findings showed that the metals determined in the inductively coupled plasma-mass spectrometry analytical results were consistent with the metal compositions of the ENDS device components that were exposed to the liquids and that nickel is a major constituent in many ENDS internal components. As a result of the exposure of the nickel-containing components to the ENDS liquids, resulting aerosol nickel concentrations per puff were higher from devices that contained lactic acid in comparison to devices with benzoic or levulinic acid. The aerosol nickel concentrations in 10 puffs from ENDS-containing lactic acid were, in some cases, hundreds of times higher than cigarette mainstream smoke nickel deliveries. Thus, the design of an ENDS device in terms of both physical construction components and the liquid chemical formulations could directly impact potential exposures to toxic constituents such as metals.

## 1. Introduction

First-generation ENDS devices were predominantly single use, shaped like cigarettes, and were sometimes called cigalikes [1]. ENDS product designs continued to advance through second- and third-generation devices [2]. Earlier generation devices with new appearances and designs are still available and popular, but fourth-generation ENDS devices now have rechargeable batteries and prefilled replaceable pods [3]. Changes in device designs may alter the overall total delivery and the relative constituents in aerosols. Therefore, it is important to examine and monitor the physical designs, liquid constituents, and aerosols emissions.

In addition to the rapid progression of ENDS designs, changes in the product’s liquid composition may also change the concentrations of aerosol constituents. Prior to the arrival of Juul^®^ products on the market, most ENDS liquids consisted of various combinations with propylene glycol, glycerol, water, nicotine, and flavors [4,5]. Now many products also include nicotine in salt form via a 1:1 molar addition of an organic acid such as benzoic acid, levulinic acid, lactic acid, and others [4,5]. In previous work, we reported that the length of time that ENDS device metal components were exposed to the liquid was a factor in the extent of corrosion that produced the metals in ENDS liquids and aerosols [6,7]. The addition of acids to ENDS liquids, however, could accelerate the corrosion of internal metal surfaces exposed to ENDS liquids. Therefore, the effect of pH on metal concentrations in aerosols was examined among pod devices with approximately the same age. In that study of metal concentrations in ENDS aerosols from three different fourth-generation pod devices, differences in the slightly acidic pH range of 4.02 to 6.79 demonstrated little or no correlation with aerosol metal concentrations from freshly obtained devices [3].

If some organic acids form multidentate ligands with metal ions and accelerate the formation of metal oxide particulates or dissolved metal concentrations compared to carboxylates that could only form monodentate ligands, the health risk from the inhalation of the aerosols with higher metal concentrations would be increased. We investigated this possibility using our validated aerosol trapping and analytical method [7] to compare aerosol metal concentrations produced by ENDS devices that have liquids containing benzoic acid versus select acids that may or may not increase ENDS liquid metal concentrations by acting as multidentate ligands and forming metal ion complexes.

## 2. Materials and Methods

### 2.1. Samples

ENDS devices (pods and disposables) were purchased in 2023 from retail outlets in the greater Atlanta area or online. Statistical analyses of previously reported results also included devices obtained in 2019. Devices were selected to represent products in which different acids were used to form the nicotine salts in the liquids. Samples were logged into our sample database and bar coded for identification and tracking. Devices were used for generating aerosol samples within 1 to 2 months after receipt or between 10 and 11 months after receipt. These products provided “new” and “aged” product sets to examine. 

### 2.2. Aerosol Collection and Preparation for Analysis

Aerosol was collected using our previously reported high metal purity fluoropolymer condensation tubing trap [7] with a Ceti8 (Cerulean, Richmond, VA, USA) aerosol “vaping” machine using the ISO standard 20768 vaping regimen (3 s 55 mL puff every 30 s with a rectangular puff profile determined at the port of the vaping machine in series with a 1000 Pa pressure drop restrictor device) [8]. Aerosol from 50 puffs was collected for each sample, dissolved, and rinsed from the condensation tubing as previously described with 3 × 8 mL 1% *v*/*v* hydrochloric acid (Veritas double distilled, GFS, Columbus, OH, USA) + 2% *v*/*v* nitric acid (Environmental grade, GFS, further purified in a PFA sub-boiling still, Savillex, Minnetonka, MN, USA). Rinses were diluted with the same acid mix to 25 mL in class A polymethylpentene volumetric flasks.

### 2.3. Sample Analysis by ICP-MS

Samples were analyzed using the instrumental parameters previously described [7] with an Agilent (Santa Clara, CA, USA) 8800 “triple quad” inductively coupled plasma-mass spectrometer (ICP-MS) with an Apex 2HF (Elemental Scientific, Omaha, NE, USA) desolvating introduction system. Earlier generation desolvating systems were optimized by default with a 140 °C spray chamber and 3 °C Peltier cooled chamber temperatures [8], but the newer system permitted software-controlled temperature optimization. We reported the optimization of spray chamber and Peltier-cooled chamber temperatures at 100 °C and 2 °C, respectively, that were used for these analyses for improved calibration and precision in the analysis of volatile forms of cadmium, zinc, and tin [9]. Five individual fully charged and previously unused devices or battery and pod combinations were used for the collection of five replicate analyses of aerosol from each brand. Metal concentrations in the five replicate analyses of aerosols from individual pods or disposable devices were reported in ng metal per 10 puffs in order to provide an approximate comparison with metal exposures in mainstream smoke from a cigarette. 

Instrument calibration was established for chromium, nickel, copper, zinc, cadmium, tin, and lead with National Institute for Standards and Technology (NIST) traceable standards from High Purity Standards (Charleston, SC, USA) prepared in 2% *v*/*v* nitric acid, 1% *v*/*v* hydrochloric acid, and 0.25% *v*/*v* ultrahigh purity hydrofluoric acid (GFS, Columbus, OH, USA). Validation procedures, determinations of limits of detection, and calibration ranges were determined as previously reported [7]. Quality Control samples were prepared as described [7] in a final volume of 25 mL in the same acid mix used for calibration preparation by adding 200 µL of 50% *v*/*v* glycerol (Sigma-Aldrich Bioultra grade, St Louis, MO, USA), 50% *v*/*v* propylene glycol (Sigma-Aldrich FCC grade, St Louis, MO, USA), and spiking near the high and low ends of the calibration range for each metal with NIST traceable standards from Inorganic Ventures (Christiansburg, VA, USA). Quality control was maintained using a modified Westgard approach with server provided SAS software (CARY, NC, USA), currently version 9.4 [10] for the analysis of high and low spike results before and after sample analyses for each analytical run. Statistical analysis of results was accomplished using JMP Pro 16 software (Cary, NC, USA).

### 2.4. Microscopy

A Thermo FEI Quanta 250 field emission gun scanning electron microscope (SEM, Hillsboro, Oregon, USA) with Oxford energy dispersive X-ray spectroscopy attachment with 80 mm^2^ X-Max silicon drift detector and Aztec software (EDS, Oxford Instruments, Concord, MA, USA) was used to identify ENDS components as possible sources for metals in the ENDS liquids and aerosols. Data were obtained in low vacuum mode at 40 Pa vapor pressure with 20 kV accelerating voltage using a large field detector. Light microscopy was performed using an Olympus (Center Valley, PA, USA) SZX16 stereo microscope with transmitted light base, DP73 camera, Cellsense operating system, and Fostec illuminator.

## 3. Results

### 3.1. ICP-MS Results

The aerosol metal (chromium, nickel, copper, zinc, cadmium tin, and lead) concentration data obtained from devices used in this study are summarized in Table 1. Cadmium concentrations in aerosols from all devices continued to be lower than the validated method detection limit (LOD) regardless of the age of the device and the type of nicotine acid salt. This is consistent with the fact that ENDS device components that could corrode and become sources of metals in ENDS liquids have not been found to be constructed from cadmium or cadmium-containing alloys. The cadmium results are also in line with validated and actively verified method limits of detection and our previously reported results [3,6,7,11]. We continued to monitor cadmium levels even though there was no cadmium in the device materials or any plausible explanation for why it would be present in the liquid contents because other research groups published values that likely resulted from background contamination from glass or low purity quartz used in sample collection or preparation [12,13,14].

Aerosol lead concentrations were also below the lowest reportable level regardless of age and type of nicotine acid salt, with only one exception. The lead concentration in aerosol from one Brez^®^ pod was above the lowest reportable level (LRL). This contrasts with previous findings [7] and is likely due to the avoidance of tin–lead solder in new products compared to the older designs. Although tin was detected in aerosols from some devices, it is likely that the concentrations were very low for the same reason. 

Copper was below the reportable level in aerosols obtained from bidistick^®^ devices analyzed within 1–2 months after purchase, although it had risen above detectable levels in aerosols analyzed within 10–11 months after purchase. In contrast, zinc concentrations were detectable but very low and not significantly different over the two timeframes. Low concentrations of copper were detected in aerosols from Brez^®^ and glas^®^ devices, but zinc concentrations were lower than the reportable level. Taken together, the low or undetectable copper and zinc concentrations suggest the absence or decreased utilization of brass components in more recent ENDS products in contrast to earlier generation devices [8]. 

Nickel and copper concentrations were slightly higher in aerosols from older NJOY Ace^®^ devices compared to the newer devices. Taken together with the copper results for bidistick^®^ devices, this provides limited support for the liquid exposure time as a factor in metal concentrations in aerosols, although the type of nicotine salt appears to be the greater factor. Nickel was the only metal that was found at concentrations greater than the method LRL in aerosols obtained from blu^®^ devices.

### 3.2. SEM-EDS Results

**Figure 1:**
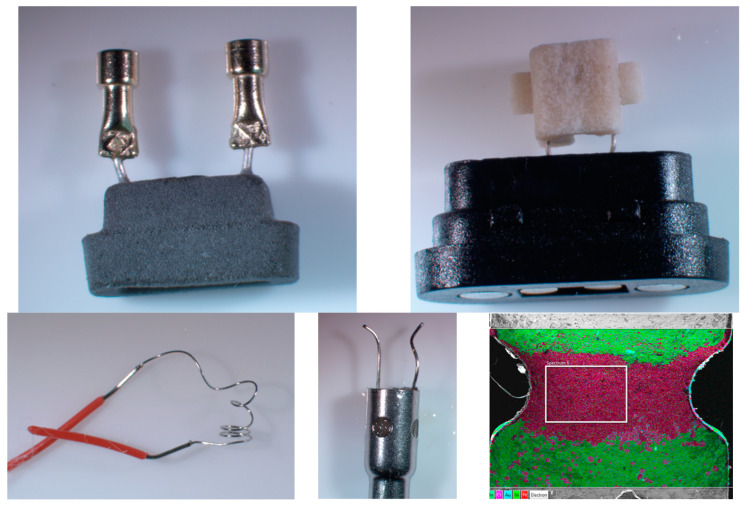
All images are CDC-sourced by the authors. (Upper left) Ceramic heating element with nickel wire and copper, zinc, and gold-coated nickel connectors from NJOY Ace^®^ pods. (Upper right) Ceramic heating element with nickel connecting wires from Brez^®^ pods. (Lower left) Bidistick^®^ nickel connecting wire fused to a heating element composed of nickel and chromium. (Lower middle) glas^®^ heating element connections extending from the housing composed of iron, chromium, nickel, and molybdenum. (Lower right) SEM-EDS image of blu^®^ electrical connector showing missing coating and exposed substrate at the bend (green is false image showing gold and nickel coating; red/violet is a combination of iron, chromium, and manganese).

The principal metal components of NJOY Ace^®^ pods that are exposed to the ENDS liquid are a ceramic heating element (silicon oxide with a smaller percentage of aluminum oxide), a nickel wire, and a nickel connector with a thin coating containing gold, copper, and zinc (Figure 1). These data are consistent with the high concentrations of nickel, copper, and zinc in the aerosol from NJOY Ace^®^ devices. Chromium was not a major constituent of any component, but possibly a minor constituent of the wires, connectors, or coatings, which is consistent with the low concentration of chromium in the aerosols from NJOY Ace^®^ pods compared to much higher nickel, copper, and zinc concentrations.

The principal metal components of Vuse Alto^®^ pods that are exposed to the ENDS liquid are a nickel wire and a nickel–gold-plated electrical connector that had small percentages of copper and zinc. A metallic heating element was predominantly embedded in a ceramic heating block (silicon oxide with a smaller percentage of aluminum oxide). A small portion of the embedded element that was exposed was predominantly nickel with smaller amounts of iron and chromium. These data are consistent with the high concentrations of nickel, copper, and zinc in aerosols obtained from Vuse Alto^®^ pods. Chromium was not a major constituent of any component, which is consistent with the low concentration of chromium in the aerosols from Vuse Alto^®^ pods.

The principal metal components of blu^®^ pods that are exposed to the ENDS liquid are a heating element composed of predominantly nickel and chromium with some iron and manganese, and an aerosol tube composed of predominantly iron and chromium with less nickel and molybdenum, likely a type of stainless steel. Electrical connectors were manufactured with a substrate of iron, chromium, nickel, and less manganese, likely from stainless steel. The coating consisted of a gold–nickel alloy, which appeared to have come off the connectors where they were apparently bent during installation, perturbing the gold coating (Figure 1). The presence of nickel in various proportions in the electrical connector coating, substrate, and all other components is consistent with the high concentrations of nickel in aerosols obtained from blu^®^ pods.

The principal metal components of bidistick^®^ disposable devices that are exposed to the ENDS liquid are a nickel connecting wire and a heating element composed of nickel and chromium, possibly nichrome (Figure 1). The aluminum case was coated with a polymer and apparently not in contact with the liquid. Since nickel–chromium alloys such as nichrome are highly resistant to elevated temperatures and oxidation [15], these data are consistent with the low concentrations of nickel and chromium in the aerosol and the small quantities of zinc and tin that are likely minor metallic impurities.

The principal metal components of Brez^®^ pods that are exposed to the ENDS liquid are a connecting wire and coil composed of iron, chromium, nickel, and zinc that pass through a ceramic heating element made of silicon oxide and a smaller percentage of aluminum oxide (Figure 1), and a vapor tube that was composed of iron, chromium, nickel, and molybdenum, in proportions consistent with an alloy such as Hastelloy that is resistant to oxidation and corrosion in the presence of acids and salts [15]. These data are consistent with the relatively low concentrations of nickel detected in aerosols from Brez^®^ pods. Copper was only present as a constituent of battery contact pins, the tips of which were exposed to the liquid. This is consistent with the low concentration of copper in the aerosols from devices. The other constituent metals of the device components were below the limits of detection.

The principal metal components of glas^®^ pods that are exposed to the ENDS liquid are a heating element connected to battery contacts and a heating element housing tube composed of iron, chromium, nickel, and molybdenum (Figure 1) consistent with Hastelloy. Nickel was the only metal among the major constituents of the device components that was present in aerosol above detection limits. Copper was only present as a constituent of battery contact pins, the tips of which were exposed to liquid, which is consistent with the low concentration of copper in the aerosols from devices.

### 3.3. Analysis of ICP-MS Results

**Figure 2:**
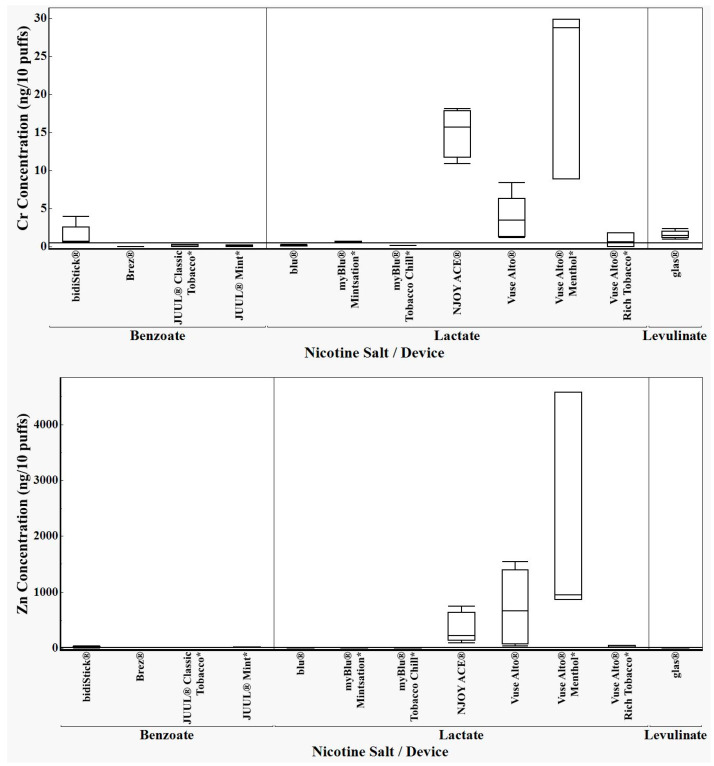

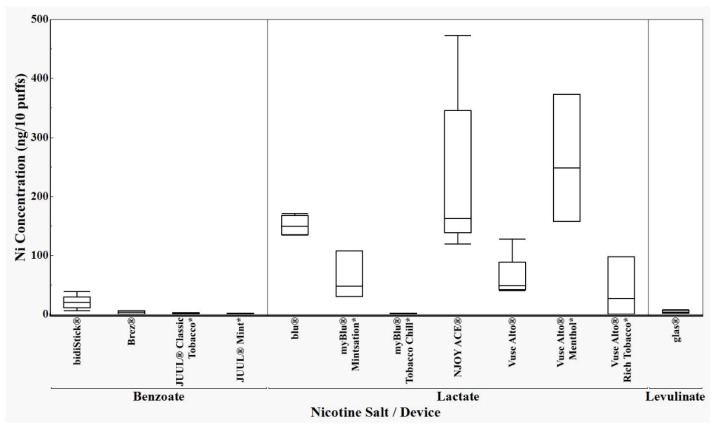
All images are CDC-sourced by the authors. Brand-dependent ENDS aerosols containing lactic acid have higher levels of chromium, nickel, and zinc than devices with other acid formulations. This statistical analysis (ANOVA) includes analyses of data previously reported^3^ as well as data reported in Table 1 to increase the number of results included in order to strengthen support for the findings beyond the devices included in the present report. Chromium concentrations in aerosols from devices that contained the lactic acid salt were significantly higher than devices that contained the benzoic acid or levulinic acid salt even when including the lower LRL blu^®^ chromium result (*p* = 0.0281). When zinc was sufficiently present in ENDS components, aerosol zinc concentrations were higher among devices that had nicotine lactic acid salts, but the means for all devices were not significantly different (*p* = 0.126). Devices that contained the nicotine lactic acid salt had significantly higher aerosol nickel levels (bottom, *p* = 0.0002) than devices that contained the benzoic acid or levulinic acid nicotine salts.

Metal concentrations in previously published aerosol data [3,6,7,11] showed a relation between storage time and aerosol metal concentrations. For most of the ENDS products analyzed in this study, there was approximately a one- to two-month period between device purchase and analysis. Data from our previous results [3] are included for comparison with this study (all new products purchased in 2023) according to time between purchase and analysis. We examined aerosols from devices containing three different nicotine salts to determine whether statistical differences in aerosol metal concentrations related to the acid used to form the nicotine salt exist. The ANOVA data presented graphically in Figure 2 clearly show differences in metal concentrations from ENDS with differing nicotine salt formulations. We found that aerosols from devices containing lactic acid nicotine salt had higher aerosol metal deliveries. For example, chromium concentrations in aerosols from devices that contained the lactic acid salt were significantly higher than devices that contained benzoic acid or levulinic acid salt, even when including the lower LRL blu^®^ chromium result (*p* = 0.0281).

The box plots (Figure 2) showing the results of chromium (top), zinc (middle) and nickel (bottom) analyses in aerosols from 2023 devices in this study (*n* = 5) combined with previously obtained data *(*n* = 3) [3].

## 4. Discussion

The results obtained continue to support corrosion of the internal device or pod components as the principal sources of metals in aerosols. The data provide limited evidence to support previous assessments of the duration of time during which the device or pod components are exposed to the nicotine-containing liquids as a factor associated with increased metal concentrations in aerosols produced by the devices [7]. To investigate this further it would be helpful for manufacturers to provide a date of manufacture and expiration dates on their products. Since analyses of the devices and pods were completed 1 to 2 months after receipt or between 10 and 11 months after receipt without knowledge of the exact date of manufacture, this represents a limitation to these findings.

In addition to the duration of exposure to components from the liquids, the data reported here provide evidence that concentrations of some metals in aerosols from different devices are significantly different during similar component liquid exposure times. A strong correlation between the type of acid used to form the nicotine salt and aerosol metal concentrations was determined, especially for nickel. This is unlikely to be due to the presence of nicotine alone. blu^®^ had 3.6% nicotine in the form of the lactate salt and bidistick^®^ had 6% nicotine in the form of the benzoate salt, yet aerosol obtained from blu^®^ pods that had the lactic acid salt had much higher nickel concentrations.

The pH is not the only factor to consider regarding whether an acid additive might accelerate the corrosion of internal ENDS metal components. The acid used in Juul^®^ products is benzoic acid, but levulinic acid [5], salicylic acid, lactic acid, malic acid, and tartaric acid [5,16], have also been reported in electronic cigarette liquids. The pKa1 values for salicylic acid, lactic acid, malic acid, levulinic acid, tartaric acid, and benzoic acid all lie between 2.95 and 3.86 [17], so none of these is a particularly strong acid. When added to a solution containing nicotine, these acids form conjugate acid/base salt buffers, resulting in the slightly acidic pH range previously reported [3]. When an organic acid carboxylate group is considered a ligand, the formation of a stable metal complex ion could accelerate the oxidation of a metal surface since the formation of the metal complex ion would shift the equilibrium away from the free ion or metal surface oxide toward the more stable ionic complex, permitting the oxidation of additional ions from the metal surface. Benzoic acid is only capable of acting as a monodentate ligand, whereas acids such as salicylic acid, lactic acid, malic acid, levulinic acid, and tartaric acid have additional oxygen-containing functional groups and are potentially capable of forming bidentate ligand complexes with metals. It is possible that the formation of stable ionic metal complexes with acids that may form bidentate ligands might accelerate the oxidation and corrosion of ENDS device metal surfaces. This would increase the concentrations of metals in the liquids from which the aerosol is generated versus monodentate acid salts or those without acid in their formulation.

Devices that contained the benzoic acid nicotine salt produced aerosols with lower metal concentrations even after a longer period of exposure of components to the liquids, indicating that nicotine itself is not a significant factor in the corrosion of device components and increasing metal concentrations in aerosols. The device that contained the levulinic acid nicotine salt also had relatively low concentrations of metals in the aerosols. Previous results have shown that pH alone is not a significant factor over the short term [3]. In that previous study, devices that contained lactic acid did not have the lowest pH. In contrast, results reported here show that the type of acid used to form the nicotine salt was a major factor in determining concentrations of metals in aerosols.

With the exception of aerosols from blu^®^ and myblu^®^, in which chromium was present predominantly in the oxidation-resistant heating element alloy resembling nichrome, devices that contained lactic acid salts of nicotine had significantly higher aerosol chromium concentrations than devices that contained the benzoic acid and levulinic acid nicotine salts (*p* = 0.0281). With the exceptions of blu^®^ and myblu^®^, in which SEM-EDS analysis determined no appreciable zinc, devices that contained lactic acid nicotine salts had significantly higher aerosol zinc concentrations than devices that contained the benzoic acid and levulinic acid nicotine salts. The overall ANOVA analyses did not show a significant difference in aerosol zinc concentrations when aerosols from all devices were included in the analyses due to the absence of significant zinc-containing components in some devices that had lactic acid salts (*p* = 0.126). Devices that contained lactic acid nicotine salt had significantly higher aerosol nickel levels (bottom, *p* = 0.0002) than devices that contained the benzoic acid or levulinic acid nicotine salts. 

**Figure 3:**
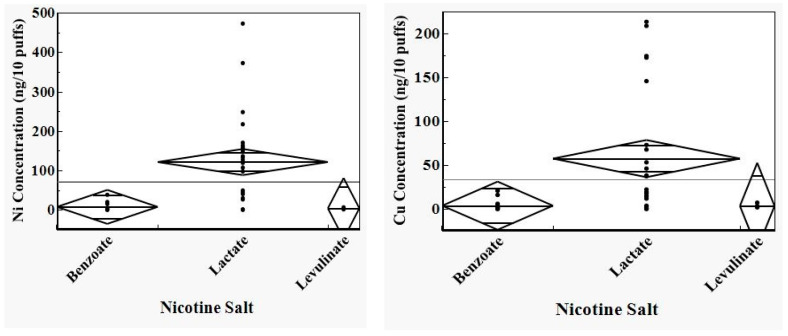
All images are CDC-sourced by the authors. Statistical analysis of the effect of nicotine salt formulations results (ANOVA) shows higher nickel (*p* = 0.0002) and copper concentrations (*p* = 0.0060) in the aerosol deliveries from ENDS with lactic acid nicotine salts compared to ENDS with benzoic acid or levulinic acid nicotine salts. The horizontal lines represent the grand mean.

Figure 3 shows one-way analyses of variance (ANOVA) plots of differences between metal concentrations in aerosol as a function of the type of acid added to the ENDS liquid to form the nicotine salt, including aerosol data from only the devices obtained and analyzed within 1 to 2 months in 2023. Even with a relatively short amount of time during which these device or pod components were in contact with the respective liquids, the nickel (*p* = 0.0002) and copper concentrations (*p* = 0.0060) in the liquids from which the aerosols were produced became significantly higher among the devices that contained lactic acid than those that contained levulinic acid and benzoic acid. Aerosol from blu^®^ devices represented the lone exception, since aerosol concentrations of all metals that were analyzed were less than LOD or less than LRL, except for nickel. Aerosol from blu^®^ devices that contained lactic acid nicotine salt nevertheless had higher nickel concentrations than aerosol from comparable devices that had benzoic acid or levulinic acid nicotine salts.

When analyzing these findings on structural bases, the capability of lactic acid to act as a possible bidentate chelating acid must be considered. Benzoic acid has a lone carboxyl group that may be used as a ligand to chelate metals. Levulinic acid has an additional carbonyl oxygen gamma to the carboxyl group that could potentially act as a second ligand. Lactic acid, however, has a hydroxyl group alpha to the carboxyl group. It is possible that acids such as lactic acid that have an oxygen atom alpha to the carboxyl group that may act as a weaker but proximal second ligand may act to solubilize and stabilize metal ions in solution and accelerate the removal of oxidized metal species from the metal surface in a manner like the mechanism by which gold is oxidized by aqua regia. Gold is not readily oxidized by nitric acid alone, but the addition of hydrochloric acid as a source of chloride ligands to coordinate and form gold ion complexes increases the rate of oxidation of gold from the surface. The data reported here support the hypothesis that products containing lactic acid increase the metal concentrations in liquids and aerosol emissions more rapidly via metal complex formation.

The increased metal concentrations in liquids that contain lactic acid versus levulinic acid and benzoic acid may suggest the potential for increased inhalation exposure to metals from ENDS containing nicotine salts of acids that may provide a second coordinating ligand alpha to the carboxyl group, resulting in higher metal corrosion rates from device components that could increase metal concentrations in the ENDS liquids. 

Since the glass or low-purity quartz filters used to quantitatively trap the particulate phase of cigarette smoke for the analysis of organic substances contribute high levels of inorganic fibrous particulates and consequently metals to the trapped particulate, electrostatic precipitation has long been used as a preferred method for quantitatively trapping the condensed particulate phase of cigarette smoke particulate, since the glass or low-purity quartz filters are used for cigarette smoke [18]. We previously reported toxic metal concentrations determined in cigarette smoke particulate using electrostatic precipitation in high-purity quartz tubes after the optimization of probe voltage to 24 kV, above which particulate mass was not increased [19]. Thus, the recovery of the smoke particulate phase in which metals are transported was quantitative. If electrostatic precipitation had also quantitatively trapped various combinations of propylene glycol, glycerol, and water in aerosols from electronic cigarettes, it would also have been the trapping technique of choice for electronic cigarette aerosols. However, our early investigations determined that the vapors produced by electronic cigarettes did not precipitate completely before passing through the electrostatic precipitation tube [7]. Since electronic cigarette aerosol recoveries varied widely when using electrostatic precipitation, it was determined that this technique would not permit quantitative aerosol recoveries for metals analysis. Therefore, we utilized a different approach for the quantitative trapping of electronic cigarette aerosol by utilizing a sufficient length of high-purity fluoropolymer tubing to permit the quantitative recovery of aerosols containing the metals [7]. Since this technique was also validated under our ISO 17025 laboratory-accredited scope of analytical methods as providing quantitative recovery of aerosol metals, it is possible to compare the recoveries of metals from cigarette smoke with the recoveries of metals from electronic cigarette aerosols.

A comparison of the nickel concentrations that were quantitatively trapped in aerosols produced by devices that have lactic acid added to form the nicotine salt using our validated aerosol trap showed that nickel was transported at much higher concentrations in 10 aerosol puffs than in mainstream smoke per cigarette [19]. This demonstrates that the choice of acid used to form nicotine salts may result in unintended differences in aerosol metal concentrations and potentially greater health risk consequences from the inhalation of aerosols from ENDS that contain liquids with other acids/component compositions.

Whereas zinc, tin, and lead from ENDS device component corrosion were previously reported to be easily dissolved in aerosols, other corrosion products including oxides of chromium, nickel, and copper were found predominantly in particle form with low dissolved metal backgrounds relative to particle peaks [20]. Although a chelating acid would increase soluble nickel concentrations, it may also liberate the nickel oxide particulate from ENDS component surfaces during the dissolution process. Indeed, aerosols from devices that had liquids with both benzoate and lactate salts of nicotine had greater particulate levels than dissolved nickel concentrations [19]. Exposures to dissolved nickel compounds are generally more directly toxic to lung tissue, whereas poorly soluble particle exposures generally cause lower immediate toxicity but impart greater carcinogenic risk [21]. Nickel and nickel compounds are described by the IARC as group 1 human carcinogens [22]. The U.S. EPA has classified nickel refinery dust (likely metallic nickel particulate) and nickel subsulfide (a poorly soluble nickel compound) as group A human carcinogens [21]. The EPA has classified nickel carbonyl (a soluble nickel compound) as a group B2 probable human carcinogen [21]. Chronic inhalation exposure to nickel also results in diminished pulmonary function, bronchitis, pulmonary inflammatory and sensitization effects including a type of asthma specific to nickel [23]. Thus, inhalation exposures to both soluble and insoluble nickel compounds should be minimized and avoided. 

The Agency for Toxic Substances Disease Registry (ATSDR) has not calculated a chronic duration daily inhalation minimum risk level (MRL) for the insoluble nickel compound particulate. However, a chronic duration inhalation MRL of 0.00001 mg/m^3^ was reported in the toxicological profile based on the respiratory effects of soluble nickel salt exposure in rats that was adjusted for intermittent exposure and dosimetrically extrapolated to humans [23]. Based on a 55 mL puff volume and the mean concentrations of aerosol total nickel reported in Table 1, mean nickel concentrations in 10 ISO standard 20768 aerosol puffs from devices that had lactic acid in the respective ENDS liquids would range from 0.112 mg/m^3^ to 0.482 mg/m^3^. All of the aerosol nickel concentrations were greater than the 0.00001 mg/m^3^ chronic inhalation MRL in the ATSDR toxicological profile for nickel [22]. Mean nickel concentrations in 10 aerosol puffs from devices that had benzoic acid or levulinic acid in the respective ENDS liquids would range from 0.0065 mg/m^3^ to 0.037 mg/m^3^ and were therefore also above the chronic inhalation MRL calculation, though much lower than in aerosols from devices that contained the lactic acid nicotine salt. Although the MRL is a minimum risk level assuming dissolved nickel compound concentrations, ENDS aerosol metals from recently obtained pod devices, with the exception of zinc, were reported to be predominantly in solid particulate form regardless of the type of nicotine salt used in the liquid [20].

At least two of the devices with liquids containing lactic acid and elevated aerosol nickel concentrations met FDA Premarket Tobacco Product FDA Applications Guidance (PMTA). Both products have the FEELM^®^ ceramic heating element technology intended to provide potential “harm reduction” [24,25]. Thus, it remains important to assess and watch for unintended consequences of formulation and design changes on a regular basis to protect users of these devices from potential for harmful exposures as the designs keep evolving.

## Figures and Tables

**Table 1:** Comparison of toxic metal concentrations in aerosols from different pods and devices on the basis of type of nicotine salt.

	Cr (ng/10 Puffs)	Ni (ng/10 Puffs)	Cu (ng/10 Puffs)	Zn (ng/10 Puffs)	Cd (ng/10 Puffs)	Sn (ng/10 Puffs)	Pb (ng/10 Puffs)
**LOD ^1^**	0.125	0.250	0.200	5.00	0.050	0.100	0.050
**LRL ^2^**	0.500	0.500	1.00	10.0	0.200	0.200	0.500
**LACTIC ACID**							
**2022 NJOY ACE^®^ Classic Tobacco, 5% nicotine pod ^3^**	5.35 ± 1.22	265 ± 182	51.4 ± 24.1	279 ± 114	<LOD	1.19 ± 0.73	<LRL
**2023 NJOY ACE^®^ Classic Tobacco 5% nicotine pod ^4^**	15.0 ± 3.1	226 ± 142	23.9 ± 28.6	353 ± 272	<LOD	1.58 ± 1.94	<LRL
**2023 Vuse Alto^®^ Rich Tobacco 5% nicotine pod ^4^**	3.75 ± 2.91	61.8 ± 36.8	104 ± 85	719 ± 672	<LOD	3.06 ± 2.86	<LRL
**2023 blu^®^ Rich Tobacco 3.6% nicotine pod ^4^**	<LRL	151 ± 16	<LRL	<LOD	<LOD	<LRL	<LOD
**BENZOIC ACID**							
**2022 bidiStick^®^ Gold Fresh Mango 6% nicotine disposable ^3^**	0.609 ± 0.360	18.3 ± 6.1	4.57 ± 8.26	13.8 ± 8.7	<LOD	1.27 ± 1.40	<LRL
**2023 bidiStick^®^ Gold Fresh Mango 6% nicotine disposable ^4^**	1.38 ± 1.48	20.4 ± 11.8	<LRL	16.3 ± 14.6	<LOD	5.59 ± 6.47	<LRL
**2023 Brez^®^ Tobacco 5% Nicotine pod ^4^**	<LOD	3.58 ± 2.74	6.12 ± 8.46	<LRL	<LOD	<LOD	1.75 ± 3.48
**LEVULINIC ACID**							
**2023 glas^®^ Signature Tobacco 5% nicotine pod ^4^**	1.56 ± 0.52	4.38 ± 2.63	3.37 ± 2.19	<LOD	<LOD	<LOD	<LRL

^1^ Method limit of detection (LOD). ^2^ Lowest reportable level (LRL). ^3^ Aerosols from devices obtained in 2022 were generated and analyzed 10–11 months later in 2023. ^4^ Aerosols from devices obtained in 2023 were generated and analyzed 1–2 months later in 2023.

## Data Availability

The original data presented in the study are included in the article; further inquiries can be directed to the corresponding author.
